# Effect of Selective 5-Hydroxytryptamine-3 Receptor and Neurokinin-1 Receptor Antagonists on Hemodynamic Changes and Arrhythmogenic Potential in Patients Receiving Chemotherapy: A Retrospective, Observational Study

**DOI:** 10.3390/jcm13030843

**Published:** 2024-02-01

**Authors:** Utku Burak Bozbulut, Tuğba Cengiz, Ahmet Özet

**Affiliations:** 1 Department of Medical Oncology, VM Medical Mersin Hospital, Mersin 33200, Turkey; ubb_burak@hotmail.com; 2 Department of Neurology, Ankara Bilkent City Hospital, Ankara 06800, Turkey; 3 Department of Medical Oncology, Ankara Gazi University, Ankara 06500, Turkey; aozet@hotmail.com

**Keywords:** chemotherapy, 5-hydroxytryptamine-3 receptor antagonists, neurokinin 1 receptor antagonists, arrhythmia

## Abstract

**Background:** Prior speculation suggests that selective 5-hydroxytryptamine-3 receptors and neurokinin-1 receptor antagonists may increase arrhythmia risk and induce electrocardiographic changes. This study examined the effect of anti-emetic medications on arrhythmogenic potential and hemodynamic alterations. **Methods:** We considered patients aged 18 or above receiving chemotherapy between June 2013 and December 2013. Patients were grouped by anti-emetic medication: intravenous granisetron (Group G), oral aprepitant plus IV granisetron (Group AG), IV palonosetron (Group P), and oral aprepitant plus IV palonosetron (Group AP). We recorded blood pressure and electrocardiography initially and at the thirtieth minute post-medication, focusing on P dispersion, QTc dispersion, and systolic/diastolic blood pressure alterations. **Results:** The study included 80 patients (20 per group). Baseline systolic/diastolic blood pressure and P dispersion showed no significant variance. However, the baseline QTc dispersion was significantly lower in Groups P and AP than G and AG. The thirtieth-minute systolic/diastolic blood pressures were significantly lower than the baseline for Groups AG and AP, and the heart rates decreased in all groups. Group P showed significantly fewer blood pressure changes. **Conclusions:** We found no arrhythmogenic potential linked to granisetron, palonosetron, and aprepitant. Hypotension was more frequent at 30 min post-medication in granisetron or aprepitant recipients. Considering no hypotension occurred when using palonosetron alone, this treatment was deemed safer.

## 1. Introduction

Chemotherapy-induced nausea and vomiting (CINV) are among the most devastating complications for cancer patients [1,2]. A number of medications have been used to prevent and treat CINV, both as stand-alone and combined treatments. Although the clinical efficacy of the selective 5-hydroxytryptamine-3 receptor antagonist (5-HT3-RA) and neurokinin-1 receptor antagonist (NK1-RA) has been demonstrated in various trials, there is controversy about their optimum dosages and combinations in preventing and treating CINV [1]. The conflicting data on the outcomes of these anti-emetic combinations between different studies might be related to differences in the 5-HT3-RA types, dosages, underlying diseases, and patients’ characteristics [1,3]. The commonly preferred anti-emetic agents are first and second-generation 5-HT3-RAs, e.g., granisetron and palonosetron, and first-generation NK1-RAs, e.g., aprepitant [3]. It is generally known that double anti-emetic therapy, including one second-generation 5-HT3-RA medication, is more effective than triple anti-emetic therapy, including one first-generation 5-HT3-RA medication, in terms of delayed nausea and vomiting [1,4,5]. Nevertheless, the relevant literature data are insufficient to prove the superiority of using new medications as a stand-alone or combined treatment over other treatment regimens in preventing and controlling CINV [6]. A number of demographic and clinical factors, including younger age, female gender, and use of anthracyclines plus cyclophosphamide or carboplatin-based chemotherapy, reportedly increase the risk for CINV [1,7,8]. Anti-emetic therapies, including 5-HT3-RAs and NK1-RAs, are also not free from complications. It has been estimated that 35 of every 1000 participants using granisetron plus aprepitant, a new generation NK-1 RA medication, will experience adverse events [6]. The incidence of adverse events varies depending on the type of medication being used. For example, 5-HT3 RAs have been questioned in terms of increasing the risk of arrhythmia and electrocardiographic changes [9,10,11]. Prolongation of the QT interval leading to the development of potentially fatal tachyarrhythmia torsades de pointes has been reported in children receiving chemotherapy [12,13,14]. Such adverse effects were usually reported in association with former 5-HT3-RAs, such as dolasetron. Nevertheless, the cardiac morbidity of new 5-HT3-RAs remains unclear [9].

The objective of this study is to investigate the arrhythmogenic potential of stand-alone or combined use of anti-emetic medications and the causal relationship of anti-emetic medications with undesirable hemodynamic changes.

## 2. Materials and Methods

### 2.1. Research Design

This retrospective study was conducted with patients aged 18 or over who received chemotherapy at the Department of Medical Oncology of Gazi University Faculty of Medicine between June 2013 and December 2013. The study protocol was approved by the local ethics committee (Gazi University, Ethical Committee for Clinical Studies, Decision Number: 259016007867, Decision Date: 25 November 2013). The study was carried out in accordance with the ethical principles set forth in the Declaration of Helsinki. Written informed consent was obtained from the patients. When the patients gave their consent for treatment during the chemotherapy phase, ICF was also taken at the same time.

### 2.2. Population and Sample

This study’s population consisted of all consecutive adult patients (aged 18 or over) treated in the Outpatient Chemotherapy Unit between June 2013 and December 2013. The files and treatments of the patients receiving treatment in the clinic were examined, and the patients were evaluated in accordance with the study inclusion criteria. Patients who had hypothyroidism (thyroid stimulating hormone level < 0.5 IU/mL), hyperthyroidism (thyroid stimulating hormone level > 4.5 IU/mL), Eastern Cooperative Oncology Group (ECOG) performance score 3 or 4, hemoglobin level < 7 g/dL, electrolyte disturbances (hypo/hypernatremia, hypo/hyperkalemia, hypo/hypercalcemia), and were receiving chemotherapy protocols were excluded from the study. All the treatments of the patients were examined; it was determined that they did not receive any anthracycline treatment during the treatment process. Additionally, patients who had previously received anthracycline group chemotherapy drugs were also excluded from the study. A total of 700 patients were evaluated; 174 were in the granisetron group, 218 in the aprepitant + granisetron group, 183 in the palonosetron group, and in the palonosetron + aprepitant group, 125 patients were examined.

### 2.3. Interventions

Normally, BP and ECG are routinely checked in our clinic. Premedication before treatment ends within 30 min. ECG and BP measurements are taken before chemotherapy begins, at the 30th minute, and hourly during treatment. An experienced nurse measured systolic blood pressure (SBP) and diastolic blood pressure (DBP) in all patients immediately before and 30 min after administering the anti-emetic medications. Concurrently, 12-lead electrocardiography (ECG) was performed on all patients. All ECGs were evaluated manually by the same cardiologist blinded to the treatment details. The ECG parameters were heart rate, P wave with the shortest (Pmin) and longest (Pmax) intervals, P dispersion (Pd), PR interval, QTc interval with the shortest (QTc-min) and longest (QTc-max) intervals, QTc dispersion (QTcd), and QRS duration [13,14].

### 2.4. Variables

The patients’ demographics (age and gender) and clinical characteristics (oncological diagnosis and comorbidities) were recorded. The laboratory tests included measurements of hemoglobin, sodium, potassium, albumin, and calcium. The hemodynamic (SBP and DBP) and ECG parameters were measured initially and at the 30th minute after administering anti-emetic medication. Since the patients’ basal systolic and diastolic blood pressures were measured, it is not important for the study if the patients had high levels before they took anti-emetic drugs. Since the BP drop below that value is evaluated after the 30th minute, it is not necessary to add a fat factor.

### 2.5. Groups

Eighty patients were divided into the following groups according to the anti-emetic medications they were administered, with each group consisting of 20 patients: (1) Group G—3 mg intravenous (IV) granisetron; (2) Group AG—125 mg oral aprepitant plus 3 mg IV granisetron; (3) Group P—250 mcg IV palonosetron; (4) Group AP—125 mg oral aprepitant plus 250 mcg IV palonosetron. All oral and IV medications were given one hour before and immediately before initiating the chemotherapeutics on the first day of the treatment courses, respectively.

### 2.6. Statistical Analysis

The study’s primary outcomes were the changes in ECG findings, Pd and QTcd values, and hemodynamic changes observed between the study’s endpoints, that is, immediately before and 30 min after administering anti-emetic medications. The descriptive statistics obtained from the collected data were expressed as the mean ± standard deviation values in the case of present continuous variables determined to conform to the normal distribution, as the median with minimum-maximum values in the case of continuous variables determined not to conform to the normal distribution, and as numbers and percentage values in the case of categorical variables. The Shapiro–Wilk, Kolmogorov–Smirnov, and Anderson–Darling tests were used to analyze the normal distribution characteristics of the numerical variables.

When comparing two independent groups, the independent samples *t*-test and Mann–Whitney U test were used in the case of numerical variables determined to conform or not to conform to the normal distribution, respectively.

When comparing more than two independent groups, one-way analysis of variance (ANOVA) and Kruskal–Wallis tests were used in the case of numerical variables determined to conform or not to conform to the normal distribution, respectively. The Fisher-Freeman-Halton test was used to compare the differences between categorical variables in RxC tables.

The Wilcoxon and Friedman tests were used in cases where two and more than two measurements were performed to examine the changes in numerical variables over different intervals, respectively.

In analyses where non-parametric tests were used, the differences between the groups were evaluated with the Games–Howell and Dwass–Steel–Critchlow–Fligner tests.

Jamovi project 2.3.18 (Jamovi, version 2.3.18, 2022, retrieved from https://www.jamovi.org, accessed on 16 April 2022) and JASP 0.16.4 (Jeffreys’ Amazing Statistics Program, version 0.16.4, 2022, retrieved from https://jasp-stats.org, accessed on 16 April 2022) software packages were used in the statistical analyses. The probability (*p*) statistics of ≤0.05 were deemed to indicate statistical significance.

## 3. Results

The study group comprised 80 patients, 20 in each anti-emetic medication group. The median age of the study group was 55 (range 19–86) years. There were 42 (52.5%) male and 38 (47.5%) female patients. The most common type of cancer in the study group was colorectal cancer (25%), followed by lung and bronchus cancer (16.3%) and breast cancer (15.0%).

There was no significant difference between the anti-emetic medication groups in terms of age, gender, and comorbidities (*p* > 0.05) (Table 1). On the other hand, the groups differed significantly in the distribution of primary tumors (*p* < 0.05). The number of colorectal and pancreatic cancer patients was significantly higher in Groups P and AP than in Groups G and AG (*p* = 0.126 and *p* = 0.012, respectively) (Table 1).

There was no significant difference between the groups in baseline laboratory parameters (*p* > 0.05) (Table 2). 

The baseline hemodynamic measurements and ECG findings are given in Table 3. There was no significant difference between the groups in SBP, DBP, and Pd values (*p* = 0.930, *p* = 0.233, and *p* = 0.062, respectively). There was a significant difference between the groups in QTcd values (*p* < 0.001). Post hoc analysis revealed that the OTcd values in Groups P and AP were significantly lower than in Groups G and AG (*p* < 0.001 for all cases). On the other hand, there was no significant difference between groups P and AP and groups G and AG in QTcd values (*p* = 0.980 and *p* = 0.780, respectively). 

There were significant differences between the baseline hemodynamic measurements and ECG findings and the hemodynamic measurements and ECG findings measured at the 30th minute after the administration of the anti-emetic medication in each group (*p* < 0.005) (Table 4). Although the SBP and DBP values measured at the 30th minute after administering the anti-emetic medication were significantly lower than the baseline SBP and DBP values in all groups, the differences between the said values reached statistical significance only in Groups AG and AP (*p* < 0.05). Although the main effect of the decrease in SBP and DBP in the AG Group was due to the aprepitant; a decrease is also observed in the granisetron iv administration. There may also be a synergistic effect. The heart rate values measured at the 30th minute after administering the anti-emetic medication were significantly lower than the baseline values in all groups (*p* < 0.05). There was no significant difference between the baseline Pd and QTcd values and the Pd and QTcd values measured at the 30th minute after administering the anti-emetic medication in any group (*p* > 0.05). The intra-group comparisons of other ECG parameters are summarized in Table 4.

The intergroup comparisons of percent changes (Δ %) in the hemodynamic measurements and the dispersion-related ECG findings are shown in Table 5. There was no significant difference between the groups in the percent changes observed in Pd and QTcd (*p* > 0.05). On the other hand, there were significant differences between the groups observed in percent changes in SBP and DBP (*p* < 0.001 and *p* = 0.005, respectively). The percent changes observed in SBP and DBP values in Group P were significantly less than in Groups AG and AP (*p* < 0.001 and *p* = 0.004, respectively, for SBP and *p* < 0.001 and *p* = 0.023, respectively, for DBP). Other post hoc comparisons did not reveal any significant difference between the groups (*p* > 0.05). 

## 4. Discussion

The findings of this study revealed that patients who received stand-alone palonosetron as the chemotherapy regimen had more stable blood pressure values than those who received granisetron and aprepitant. Additionally, there was no significant difference between the groups ‘anti-emetic ECG findings in terms of arrhythmogenic potential. Patients’ premedication is administered 30 min before chemotherapy. This period was chosen because the chemotherapy regimens themselves will also have an effect if we look at the 60th and 90th minutes.

The correlation between the changes in ECG parameters and 5-HT3-RAs has been addressed in the literature, particularly in healthy volunteers, yet not sufficiently in cancer patients treated via chemotherapeutics [11,12,15]. In 2003, Navari et al. [11] reported small, irreversible, and clinically insignificant ECG changes and no severe cardiac adverse events in healthy volunteers and patients who received 5-HT3-RAs and underwent chemotherapy or surgery. Pinarli et al. [13] reported a significant shortening of the PR interval and QRS complex durations between the 90th minute and 24th hour after granisetron use in children with solid tumors. Significant prolongation of the QTc and the shortest QTc interval between the 60th minute and 90th minute after granisetron use was another ECG change observed in pediatric oncological patients [12,13]. The findings reported in the literature on the ECG changes observed after administering 5-HT3-RAs are contradictory [12,16,17]. Recent studies did not report any significant cardiac adverse events in oncological patients using anti-emetic medications [1,2,3,4,5,7,8,18,19,20]. Taken together, these data suggest that the newly developed and new-generation anti-emetic medications with higher safety and efficacy features may not lead to cardiac adverse events.

The ECG changes were reportedly the most prominent within one and two hours after 5-HT3-RA administration [11,12]. In this study, ECG was performed 30 min after administering the anti-emetic medication. This relatively shorter interval might have resulted in the detection of premature ECG changes in the groups. Nevertheless, these ECG changes were most likely clinically insignificant without any sequelae.

Sinus bradycardia was another complication reported, albeit rare, following the administration of 5-HT3-RA [21]. Buyukavci et al. [12] reported significant decreases in the mean heart rate one and three hours after administering the anti-emetic medication in children using granisetron. In comparison, significant decreases in heart rate were observed in all groups included in this study after administering anti-emetic drugs, although not low enough to be defined as bradycardia. Similar findings were reported in the literature [21]. Hence, bradycardia might be considered a transient, self-limiting event with clinical insignificance.

The arrhythmogenic potential of several 5-HT3-RAs, including dolasetron, ondansetron, and granisetron, have been addressed in the literature [10,22]. QTc prolongation of less than 15 msec was another common finding in patients using 5-HT3-RAs [11]. Granisetron caused significant QTd and QTcd prolongations in pediatric oncology patients at the first hour of its infusion [12]. These prolongations were regarded as transient changes lacking any clinical significance [11,12]. Tricco et al. [9] investigated the safety and effectiveness of different serotonin receptor antagonists in chemotherapy patients in a systematic review and network meta-analysis. They found no significant difference between the serotonin receptor antagonists in terms of arrhythmia and mortality. They reported that the risk for QTc prolongation was significantly higher in patients who received dolasetron plus dexamethasone. Based on this systematic review, granisetron and palonosetron can be deemed safer than other agents in terms of cardiac morbidity [9]. In comparison, in this study, only limited increases (often less than 10 msec) were detected in QTc parameters. In addition, these ECG changes did not lead to any clinical consequences. Therefore, it can be speculated that severe cardiac side effects rarely occur after administering anti-emetic drugs in cancer patients and even more rarely in patients taking new-generation drugs. However, factors such as multi-drug chemotherapeutics and anti-emetics, drug–drug interactions, and comorbidities should be considered when evaluating OTc prolongation in cancer patients.

There is no evidence of the arrhythmogenic potential of an aprepitant. As a matter of fact, as in this study, Marbury et al. [14] did not detect QTc prolongation in healthy subjects using fosaprepitant, a water-soluble pro-drug of aprepitant.

Orthostatic hypotension is one of the most frequent adverse events induced by anti-emetic agents [10]. Ondansetron might be responsible for rare and mild hypotensive attacks, contrary to palonosetron [23]. Aogi et al. [24] and others [25,26] reported that palonosetron was not associated with hypotensive attacks that occur after its administration. Although it was scarce, chest pain with hypotension was reported in one patient using netupitant and palonosetron [19]. Uchida et al. [27] reported hypertension in 6.3% and 2.4% of the patients using palonosetron and granisetron, respectively. In comparison, hypotensive blood pressure values were observed in patients in groups G, AG, and AP after administering the anti-emetic medication. Given that there is no data on the hypotensive effect of granisetron and aprepitant in the literature, the relevant finding of this study may be the first evidence in the literature on this subject. However, considering that dehydration, electrolyte disturbances, and malnutrition are commonly encountered in chemotherapy patients, prospective studies are needed to shed more light on the subject.

## 5. Limitations

The fact that this study was not designed as a randomized study might be considered its primary limitation. In addition, the inclusion of all consecutive patients in the study, resulting in a study group with heterogeneous patient and tumor characteristics, might be deemed another limitation of the study.

## 6. Conclusions

In conclusion, no arrhythmogenic potential was detected due to any anti-emetic medication, including granisetron, palonosetron, and aprepitant, regardless of whether they were used as a stand-alone or combination therapy, in cancer patients receiving chemotherapy. Hypotension was detected more frequently at the thirtieth minute after administering anti-emetic medication in patients who received granisetron or aprepitant. Given that it did not cause hypotension, stand-alone use of palonosetron alone was considered safer. Prospective large-scale studies that stratify patient and tumor characteristics are needed to clarify the controversial issues.

## Figures and Tables

**Table 1:** Demographic and clinical characteristics of the study groups.

		Groups			
	Group G (*n* = 20)	Group AG (*n* = 20)	Group P (*n* = 20)	Group AP (*n* = 20)	*p*
Age (year) ^†^	58.0 ± 13.0	54.5 ± 12.7	51.0 ± 13.9	54.0 ± 10.8	0.466
Sex ^‡^					
Female	10 (50.0)	8 (40.0)	10 (50.0)	10 (50.0)	0.896
Male	10 (50.0)	12 (60.0)	10 (50.0)	10 (50.0)	
Primary tumor ^‡^					
Lung	1 (5.0)	5 (25.0)	2 (10.0)	5 (25.0)	0.198
Breast	5 (25.0)	3 (15.0)	1 (5.0)	3 (15.0)	0.427
Colorectal	5 (25.0)	0 (0.0)	16 (80.0)	1 (5.0)	0.126
Pancreas	0 (0.0)	1 (5.0)	1 (5.0)	6 (30.0)	0.012
Head-neck	2 (10.0)	3 (15.0)	1 (5.0)	2 (10.0)	0.956
Mesenchymal tumor	1 (5.0)	0 (0.0)	3 (15.0)	1 (5.0)	0.396
Ovary	1 (5.0)	2 (10.0)	1 (5.0)	0 (0.0)	0.900
Malignant melanoma	1 (5.0)	2 (10.0)	0 (0.0)	0 (0.0)	0.609
Others (Lymphoma. CNS. testes. urinary bladder)	5 (25.0)	4 (20.0)	0 (0.0)	2 (10.0)	0.089
Comorbidities ^‡^					
Hypertension	2 (10.0)	2 (10.0)	2 (10.0)	3 (15.0)	0.999
Diabetes mellitus	1 (5.0)	1 (5.0)	3 (15.0)	2 (10.0)	0.832
Coronary artery disease	0 (0.0)	0 (0.0)	1 (5.0)	0 (0.0)	0.999

^†^: mean standard deviation, ^‡^: *n* (%). Group G: Intravenous (IV) granisetron, Group AG: oral aprepitant + IV granisetron, Group P: IV palonosetron, Group AP: oral aprepitant + IV palonosetron. CNS: central nervous system.

**Table 2:** Laboratory investigations in the groups.

		Groups			
	Group G (*n* = 20)	Group AG (*n* = 20)	Group P (*n* = 20)	Group AP (*n* = 20)	*p*
Hemoglobin (g/dL) ^§^	12.03 (8.8–14.5)	11.29 (8–15.9)	11.97 (9.2–15.3)	11.77 (8.6–15.6)	0.539
Sodium (mEq/L) ^§^	139.85 (135–145)	138.75 (135–143)	138.55 (135–144)	139.65 (135–144)	0.313
Potassium (mEq/L) ^§^	4.56 (3.7–5.7)	4.45 (3.6–5.3)	4.26 (3.7–5.1)	4.48 (4–5)	0.223
Calcium (mg/dL) ^§^	8.91 (7.3–10.1)	9.11 (7.5–10)	9.07 (7.9–10.2)	9.23 (6.11–10.6)	0.363
Albumin (g/dL) ^§^	3.79 (2.5–4.8)	3.74 (2.2–4.6)	4.01 (2.9–4.6)	3.87 (2.7–4.5)	0.505
TSH (IU/dL) ^§^	1.92 (0.96–3.59)	2.2 (0.92–3.67)	2.03 (1.09–3.48)	2.07 (1.04–3.92)	0.605

^§^: median (min-max) Group G: Intravenous (IV) granisetron, Group AG: oral aprepitant + IV granisetron, Group P: IV palonosetron, Group AP: oral aprepitant + IV palonosetron. TSH: thyroid stimulating hormone.

**Table 3:** Comparison of the baseline hemodynamic and ECG findings between the groups.

		Groups			
	Group G (*n* = 20)	Group AG (*n* = 20)	Group P (*n* = 20)	Group AP (*n* = 20)	*p*
SBP (mmHg) ^§^	122.5 (110–140)	123.5 (110–140)	121.5 (110–130)	123 (110–130)	0.930
DBP (mmHG) ^§^	74.5 (60–80)	77 (70–90)	76 (70–80)	78 (70–80)	0.233
P_d_ (msec) ^§^	40.05 (25–48)	34.5 (22–48)	37.1 (26–56)	34.6 (21–50)	0.062
QT_cd_ (msec) ^§^	59.53 (31.1–81.4)	62.48 (20.3–79.3)	36.595 (12.6–51.4)	35.285 (20–45.1)	<0.001

^§^: median (min-max) Group G: Intravenous (IV) granisetron, Group AG: oral aprepitant + IV granisetron, Group P: IV palonosetron, Group AP: oral aprepitant + IV palonosetron. SBP: systolic blood pressure, DBP: diastolic blood pressure.

**Table 4:** Comparison of the percentage changes (Δ%) between the post-emetic 30th-minute evaluation and the baseline measurements in SBP, DBP, Pd, and QTcd between the groups.

		Groups			
	Group G (*n* = 20)	Group AG (*n* = 20)	Group P (*n* = 20)	Group AP (*n* = 20)	*p*
Δ SBP (%) ^§^	−4.94 (−15.38–9.09)	−7.88 (−14.29–0)	−1.57 (−8.33–0)	−7.49 (−15.38–9.09)	<0.001
Δ DBP (%) ^§^	−2.2 (−12.5–16.67)	−6.81 (−14.29–14.29)	0.8 (−12.5–14.29)	−5 (−12.5–0)	0.005
Δ P_d_ (%) ^§^	11.21 (−13.04–69.23)	11.03 (−31.25–60.71)	3.7 (−51.79–51.61)	4.56 (−40.91–63.64)	0.800
Δ P_d_ (msec) ^†^	3 ± 1.68	3.3 ± 1.65	0.75 ± 2.6	0.05 ± 2.1	0.700
Δ QT_cd_ (%) ^§^	0.77 (−38.47–71.12)	−7.97 (−34.39–62.56)	12.27 (−37.55–250)	4.3 (−55.53–96.58)	0.464
Δ QT_cd_ (msec) ^†^	1.7 ± 3.57	7.4 ± 2.45	−0.95 ± 2.37	0.76 ± 2.88	0.160

^§^: median (min-max), ^†^: mean standard deviation Group G: Intravenous (IV) granisetron, Group AG: oral aprepitant + IV granisetron, Group P: IV palonosetron, Group AP: oral aprepitant + IV palonosetron. SBP: systolic blood pressure, DBP: diastolic blood pressure.

**Table 5:** Comparison of the baseline and anti-emetic 30th minute hemodynamic and ECG findings in the groups.

	Groups											
	Group G			Group AG			Group P			Group AP		
	Baseline	Anti-Emetic 30th min	*p*	Baseline	Anti-Emetic 30th min	*p*	Baseline	Anti-Emetic 30th min	*p*	Baseline	Anti-Emetic 30th min	*p*
Hemodynamic parameters ^§^												
SBP (mmHg)	122.5 (110–140)	116.5 (110–130)	0.163	123.5 (110–140)	113.5 (100–120)	<0.001	121.5 (110–130)	119.5 (110–130)	0.072	123 (110–130)	113.5 (110–120)	<0.001
DBP (mmHg)	74.5 (60–80)	72.5 (70–80)	0.568	77 (70–90)	71.5 (60–80)	0.001	76 (70–80)	76.5 (70–80)	0.577	78 (70–80)	74 (70–80)	0.002
ECG findings ^§^												
Heart rate (beat/min)	84.25 (69–96)	81.9 (67–97)	0.002	78.85 (67–94)	71.3 (64–87)	<0.001	78.5 (68–90)	76.8 (65–86)	<0.001	75.9 (68–84)	68.5 (62–80)	<0.001
P_max_ (msec)	108.8 (92–118)	108.71 (71–118)	0.971	108.8 (89–116)	100.65 (65–118)	0.069	109.2 (102–118)	108.1 (74 –119)	0.364	107.7 (94–119)	102.95 (69–120)	0.073
P_min_ (msec)	68.25 (62–74)	69.65 (62–115)	0.064	70.25 (64–78)	78.65 (63–117)	0.182	72.55 (60–78)	74.6 (60–102)	0.738	71.55 (63–78)	77.7 (63–117)	0.151
P_d_ (msec)	40.05 (25–48)	43.35 (32–54)	0.071	40.05 (22–48)	37.8 (22–51)	0.061	37.1 (26–56)	36.4 (27–47)	0.78	34.6 (21–50)	34.6 (24–45)	0.981
PR interval (msec)	140.6 (125–157)	142.6 (127–158)	0.09	143.6 (127–165)	146.25 (127–165)	0.301	140.3 (124–156)	144 (134–157)	0.07	143.25 (132–156)	136.2 (123–159)	0.014
QRS duration (msec)	75.15 (60–92)	80.55 (67.5–95)	0.064	77.5 (67–93)	77.1 (63–87)	0.882	75.45 (65–96)	78.4 (67–87)	0.217	76.25 (63–89)	71.7 (62–89)	0.057
QT_cmin_ (msec)	389.27 (372.3–414.2)	399.26 (372.6–413.6)	0.004	389.27 (352.1–400.7)	401.19 (367–414)	0.08	345.79 (320.2–361.3)	350.4 (325.2–366.4)	0.13	348.36 (344.1–360.1)	353.97 (321.2–372.2)	0.069
QT_cmax_ (msec)	449.79 (428.6–473.6)	456.64 (435.7–471.7)	0.639	449.79 (372.4–470.6)	456.28 (400.2–473.2)	<0.001	382.35 (361.4–400.5)	388.24 (365.2–398.6)	0.302	383.64 (362.4–397.9)	388.52 (367.2–399.3)	0.059
QT_cd_ (msec)	59.53 (81.4–31.1)	57.83 (42.7–85.9)	0.568	59.53 (20.3–79.3)	55.08 (33–82.5)	0.07	36.6 (22.6–51.4)	37.55 (23.7–47.1)	0.694	35.3 (20–45.1)	34.53 (18.1–53.2)	0.796

^§^: median (min-max) Group G: Intravenous (IV) granisetron, Group AG: oral aprepitant + IV granisetron, Group P: IV palonosetron, Group AP: oral aprepitant + IV palonosetron. ECG: electrocardiogram, SBP: systolic blood pressure, DBP: diastolic blood pressure.

## Data Availability

If required, our data can be submitted.
